# Medical Jurisprudence

**Published:** 1861-11

**Authors:** 


ART. X.—*Medical Jurisprudence.* By ALFRED SWAINE TAYLOR, M.D., F.R.S., etc. Fifth American from the seventh and revised London edition. Edited, with additions, by EDWARD HARTSHORNE, M.D., etc. 8vo , pp. 714. Philadelphia: Blanchard & Lea, 1861.

THE merits of this valuable work are sufficiently attested by the fact that it has passed through so many editions, both in the United States and Europe. The well-known ability and industry of Dr. Taylor are sufficient guarantee, says the preface, that he has made full use of his opportunities to introduce in the last edition the latest results of legal and scientific investigations; and we may add, also, our own testimonial of appreciation of the great service to the literature of Medical Jurisprudence conferred by the author in this comprehensive and useful work. As an authority on the subject, it is perfectly reliable; and as a guide to difficult points of forensic medicine, it has superior claims on the attention of practitioner and student.

